# Anti-Inflammatory Effects of Aptamin C in Pulmonary Fibrosis Induced by Bleomycin

**DOI:** 10.3390/ph17121577

**Published:** 2024-11-24

**Authors:** Seulgi Shin, Hyejung Jo, Tomoyo Agura, Seoyoun Jeong, Hyovin Ahn, Soyoung Pang, June Lee, Jeong-Ho Park, Yejin Kim, Jae Seung Kang

**Affiliations:** 1 Laboratory of Vitamin C and Antioxidant Immunology, Department of Anatomy and Cell Biology, Seoul National University College of Medicine, Seoul 03080, Republic of Korea; seulgi.shin@snu.ac.kr (S.S.); luv_jo@snu.ac.kr (H.J.); tomoyoagura@snu.ac.kr (T.A.); jsy9804@snu.ac.kr (S.J.); jahb1220@snu.ac.kr (H.A.); banggri@snu.ac.kr (S.P.); bbambaya921@snu.ac.kr (Y.K.); 2 Institute of Allergy and Clinical Immunology, Seoul National University Medical Research Center, Seoul 08826, Republic of Korea; 3 Department of Research and Development, N Therapeutics Co., Ltd., Seoul 08813, Republic of Korea; 4 Nexmos, Inc., Yongin-si 168267, Republic of Korea; augustine@nexmos.com (J.L.); pjh01@nexmos.com (J.-H.P.); 5 Department of Applied Bioengineering, Graduate School of Convergence Science and Technology, Seoul National University, Seoul 08826, Republic of Korea

**Keywords:** Aptamin C, vitamin C, inflammation, pulmonary fibrosis, SVCT

## Abstract

**Background/Objectives**: Vitamin C is a well-known antioxidant with antiviral, anticancer, and anti-inflammatory properties. However, its therapeutic applications are limited by rapid oxidation due to heat and light sensitivity. Aptamin C, which employs aptamers to bind vitamin C, has demonstrated enhanced stability and efficacy. This study investigates the potential of Aptamin C to inhibit the progression of pulmonary fibrosis, a prominent inflammatory lung disease with no effective treatment. **Methods**: Mice bearing bleomycin-induced pulmonary fibrosis were administered vitamin C or Aptamin C, and their weight changes and survival rates were monitored. Inflammatory cell infiltration was assessed in the bronchoalveolar lavage fluid (BALF), and the degree of alveolar fibrosis was measured by H&E and Masson’s trichrome staining. To elucidate the mechanism of action of Aptamin C, Western blot analysis was performed in HaCaT and lung tissues from bleomycin-induced pulmonary fibrosis mice. **Results**: The Aptamin C-treated group showed a notably higher survival rate at 50%, whereas all subjects in the vitamin C-treated group died. Histological examination of lung tissue showed that inflammation was significantly suppressed in the Aptamin C-supplemented group compared to the vitamin C-supplemented group, with a 10% greater reduction in cell infiltrations, along with noticeably less tissue damage. Additionally, it was observed that Aptamin C increased SVCT-1 expression in the HaCaT cells and the lung tissues. **Conclusions**: Taken together, Aptamin C not only increases the stability of vitamin C but also induces an increase in SVCT-1 expression, facilitating greater vitamin C absorption into cells and tissues, thereby inhibiting the progression of symptoms and associated inflammatory responses in pulmonary fibrosis.

## 1. Introduction

Vitamin C, known as ascorbic acid, is a potent antioxidant that plays diverse roles within the human body [1,2]. It supports the function of the immune system and is crucial in aiding the growth and repair of tissues [3]. Furthermore, vitamin C enhances the absorption of iron and aids in the synthesis of collagen [4], essential for maintaining the health of skin, bones, and connective tissues [5,6]. We have been conducting studies on the antiviral, anticancer, and anti-inflammatory effects of vitamin C in recent years [7]. The anti-inflammatory efficacy of vitamin C is closely related to its antioxidant properties. Antioxidants neutralize free radicals in the body, reducing cell damage and suppressing inflammatory responses [8]. Inflammation is considered to be the cause of many chronic diseases, and the intake of vitamin C has the potential to reduce such inflammation [9]. Vitamin C is known to regulate the production of pro-inflammatory cytokines including IL-6, IL-18, and IL-22, and effectively inhibit the occurrence and progression of several kinds of inflammatory diseases, such as hepatitis, colitis, and pneumonia [10,11,12]. Studies indicate that vitamin C exhibits a high level of anti-inflammatory effects and may aid in the treatment and prevention of specific inflammatory diseases. Therefore, the anti-inflammatory efficacy of vitamin C holds many potential benefits, especially in managing inflammatory diseases such as pulmonary fibrosis.

Pulmonary fibrosis is a disease where lung tissue becomes damaged and gradually hardens, interfering with the normal function of the lungs and causing symptoms like breathlessness, persistent cough, fatigue, and weight loss [13]. The global incidence and prevalence rate of pulmonary fibrosis varies by region and population characteristics, estimated at roughly 0.09–1.3 and 0.33–4.51 cases per 10,000 people [14]. The causes of pulmonary fibrosis are diverse, with identifiable causes including long-term smoking, certain occupational exposures (e.g., asbestos, silica dust, metal vapors, side effects of drugs, radiation therapy), and other diseases (e.g., rheumatoid arthritis, scleroderma) [15]. However, most cases are classified as idiopathic pulmonary fibrosis (IPF), where the cause is unknown. Treatment depends on the cause of fibrosis, the degree of progression, and the patient’s overall health condition [16]. Currently, there is no cure for pulmonary fibrosis, and treatments primarily aim to alleviate symptoms and slow the disease’s progression [17]. Medications such as antifibrotics (e.g., pirfenidone, nintedanib) are used to inhibit the progression of lung fibrosis [18,19,20]. Additionally, oxygen therapy, pulmonary rehabilitation programs, anti-inflammatory drugs, and immunosuppressants are utilized for symptom management [21]. In very advanced cases, lung transplantation may be the only treatment option, depending on the patient’s condition and eligibility for transplantation [22,23].

Aptamers are single-stranded DNA or RNA oligonucleotides with high affinity and specificity for their target molecules [24]. They can selectively bind to specific targets both inside and outside the body, making them useful in diagnostics, therapeutic development, and biological research [25]. Aptamers are particularly valuable due to their high binding specificity, adjustable biocompatibility, and relatively low immunogenicity [26,27]. An example of an aptamer-based therapeutic is Macugen (pegaptanib), used to treat age-related macular degeneration (AMD) [28]. This drug specifically binds to the vascular endothelial growth factor (VEGF) contributing to vision loss in AMD, inhibiting its function [29,30]. Thus, Macugen suppresses abnormal blood vessel growth and reduces macular edema, slowing vision loss in AMD patients. Aptamers binding specifically to vitamin C can effectively inhibit the rapid oxidation of vitamin C, neutralizing free radicals and preventing cell damage more effectively [31]. In other words, aptamers form a protective layer around vitamin C, preventing it from reacting with oxygen or other oxidizing agents, enhancing the stability of vitamin C, and maintaining its antioxidant effects over an extended period. This maximizes the health benefits of vitamin C, including its anti-inflammatory, antiviral, and skin-protective properties. The complex of aptamers and vitamin C, termed Aptamin C, could be utilized in developing more stable vitamin C formulations, such as injectables or oral supplements [32].

Research on the efficacy of vitamin C in preventing the occurrence of pulmonary fibrosis and alleviating its symptoms has not been extensively conducted to date, but some studies suggest that vitamin C has the potential to slow the progression of pulmonary fibrosis or reduce inflammation [33,34]. For example, supplementation with vitamin C in animal models has been reported to decrease lung inflammation and fibrosis [35]. However, more research is needed before these findings can be directly applied to humans. Early-stage results from human studies also suggest that the anti-inflammatory and antioxidant effects of vitamin C could help manage symptoms in pulmonary fibrosis patients [36].

In our previous research, we found that Aptamin C is more effective than vitamin C in suppressing the inflammatory response induced by house dust mite (HDM) extracts [24]. This anti-inflammatory effect confirms the potential of Aptamin C in inhibiting skin inflammation more efficiently than vitamin C alone. Therefore, in this study, we aimed to assess the efficacy of Aptamin C in alleviating symptoms of pulmonary fibrosis by administering vitamin C and Aptamin C to animal models induced with pulmonary fibrosis using bleomycin and evaluating the extent of inhibition of pulmonary fibrosis progression.

## 2. Results

### 2.1. Aptamin C Has an Effective Inhibitory Effect on Bleomycin-Induced Pulmonary Fibrosis Compared to Vitamin C

**Figure 1:**
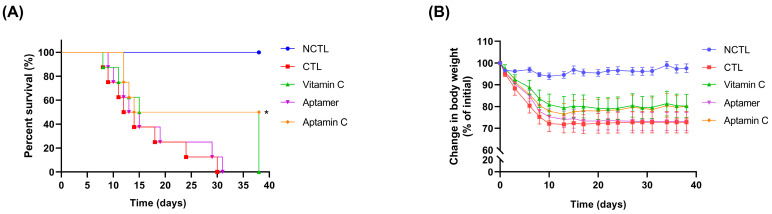
Protective effect of Aptamin C on bleomycin-induced mortality in mice. Survival rates were measured in mice (*n* = 8) subjected to intratracheal injection of bleomycin (1.5 mg/kg) or PBS alone (negative control (NCTL)) in a 30 μL total volume. The mice were intraperitoneally administered with PBS containing 1 mM of MgCl_2_ (Vehicle; Control (CTL)), 100 mg/kg vitamin C, 2 mg/kg aptamer, or a combination of vitamin C (100 mg/kg) and aptamer (2 mg/kg; Aptamin C). (**A**) Body weight change was recorded three times per week. (**B**) Mortality was monitored daily for 38 days and the percent survival rate was expressed as Kaplan–Meier survival plots. The survival plots were analyzed using a log-rank (Mantel–Cox) test. * *p* < 0.05 compared to the control.

Our previous study using Gulo (−/−) mice, which are deficient in vitamin C due to a knockout of the gulono-lactone oxidase gene, reported an increased vitamin C level in each organ at 4 weeks compared to 2 weeks after administration of vitamin C in its conventional form. Interestingly, after 4 weeks of administration, the groups treated with Aptamin C exhibited higher vitamin C levels in each organ compared to those given vitamin C [37]. Based on the results regarding vitamin C concentration in the lungs from our previous report, the efficacy of Aptamin C, particularly its anti-inflammatory properties, was confirmed for pulmonary fibrosis. Pulmonary fibrosis is a representative inflammatory lung disease, for which there are currently no effective treatments for its onset or progression. To this end, a bronchotomy was performed on C57BL/6 mice, and bleomycin (BLM) was administered intratracheally to induce pulmonary fibrosis. Vitamin C or Aptamin C was then administered daily for 5 weeks, and the survival of mice was monitored. The results showed a difference in survival rates between the vitamin C and Aptamin C groups starting from day 14. By day 38, just before the end of the experiment, all mice in the vitamin C group had died, whereas Aptamin C showed a 50% survival rate (Figure 1A). During the experimental period, weight loss in both the vitamin C and Aptamin C groups over 5 weeks was lower than in the control group, with no significant differences between the two treatment groups (Figure 1B). These results indicate that Aptamin C administration prolonged survival and reduced weight loss in mouse models of BLM-induced pulmonary fibrosis.

### 2.2. Aptamin C Effectively Inhibits the Inflammatory Response Associated with Bleomycin-Induced Pulmonary Fibrosis

**Figure 2:**
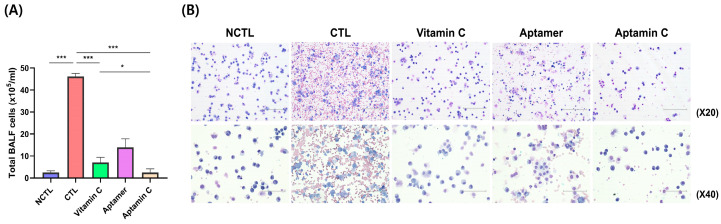
Reduced infiltration of inflammatory cells in the lungs of Aptamin C-treated mice. Gulo KO mice (*n* = 5) injected with bleomycin intraperitoneally were administered PBS containing 1 mM of MgCl_2_ (Vehicle; Control (CTL)), 100 mg/kg vitamin C, 2 mg/kg aptamer, or a combination of Vitamin C (100 mg/kg) and aptamer (2 mg/kg; Aptamin C) for 14 days. BALF was obtained by intratracheal injection of 0.8 mL PBS, followed by withdrawal twice. (**A**) The total number of BALF cells was measured using a hemocytometer following staining with trypan blue. Data are presented as the mean ± SEM. * *p* < 0.05, *** *p* < 0.001. (**B**) Representative images are shown from Wright–Giemsa stained immune cells in BALF. Photomicrographs were captured at 200× and 400× magnification (scale bar = 150 and 300 µm, respectively). Negative control, NCTL.

**Figure 3:**
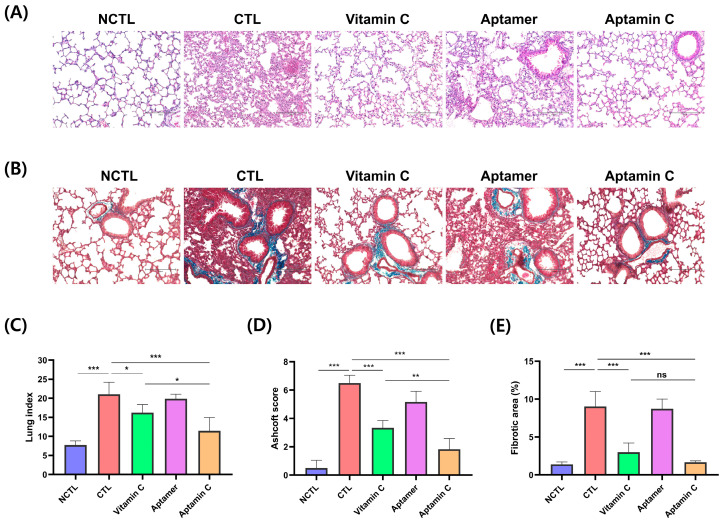
Protective effect of Aptamin C on bleomycin-induced pulmonary fibrosis. Gulo KO mice (*n* = 5) were treated with vehicle, vitamin C, aptamer, and Aptamin C for 14 days after bleomycin instillation. The mice were sacrificed, and lung tissue excised. The lung tissue sections were prepared and stained with (**A**) hematoxylin and eosin (H&E) staining and (**B**) Masson’s trichome staining. Photomicrographs were captured at 200× magnification (Scale bar = 150 µm). (**C**) The lung tissues index (tissue weight/body weight) was calculated. (**D**) The severity of lung fibrosis was assessed using the Ashcroft score based on H&E staining. (**E**) Collagen-positive areas (stained blue) based on Masson’s trichrome staining were quantified using Celleste™ image analysis software. A one-way ANOVA with Tukey’s multiple comparisons test was performed. * *p* < 0.05, ** *p* < 0.01, *** *p* < 0.001, ns: not significant. Negative control, NCTL; Control, CTL.

To confirm the effect of Aptamin C on inflammation in BLM-induced pulmonary fibrosis, Gulo (−/−) mice deficient in vitamin C for 3 weeks were administered with BLM, followed by Aptamin C therapy for 2 weeks. It is known that depending on the dose of BLM administered, pulmonary fibrosis symptoms accompanied by an inflammatory response typically become apparent two weeks after BLM administration. Therefore, immediately following BLM administration and for the subsequent two weeks, both Aptamin C and vitamin C were administered daily. After this period, the following were measured: (1) the number of inflammatory cells in the bronchoalveolar lavage fluid (BALF), (2) the weight of the lungs, (3) the degree of inflammation in the lung tissue through H&E staining, and (4) the examination of fibrotic lesions in the lungs through Masson’s trichrome staining. As shown in Figure 2, this result shows inhibition of infiltration of inflammatory cells in the BALF obtained from the lung tissues. BLM increased the number of infiltrations of inflammatory cells in the BALF, and groups administered vitamin C, aptamer, and Aptamin C decreased these cell infiltrates. Compared to the group administered BLM, vitamin C, aptamer, and Aptamin C administration reduced infiltrations of inflammatory cells by 84%, 69%, and 94%, respectively. Furthermore, an examination of the changes in lesions within the lung tissue showed that the inflammatory response was more effectively inhibited in the groups administered BLM followed by Aptamin C and vitamin C (Figure 3A,B). A significant increase in weight, indicative of edema, was observed in the lung tissue of mice administered BLM, and this increase appeared to be reduced in mice treated with Aptamin C or vitamin C (Figure 3C). The lung index was displayed at 7.73 ± 0.98 and 21.06 ± 2.80 in the vehicle and control groups. In contrast, the groups administered vitamin C, aptamer, and Aptamin C showed a reduced lung index of 16.22 ± 1.88, 19.87 ± 1.09, and 11.46 ± 3.12, respectively. The treatment of Aptamin C most effectively reduced lung fibrosis despite the administration of BLM, as evidenced by the lowest Ashcroft scale in lung tissue. Specifically, the Ashcroft scale was 3.54-fold lower than that of the control group. In comparison, the decrease observed in the group administered vitamin C was 1.95-fold lower (Figure 3D). Consistent with the results for histological fibrosis score, the fibrotic area decreased in the groups treated with vitamin C and Aptamin C compared to the control group. However, the most substantial decrease was observed in the Aptamin C-administered group. Specifically, compared to the control, the Masson’s trichome-stained area in lung tissue decreased by 66.8% and 81.4% in the groups administered vitamin C and Aptamin C, respectively (Figure 3E). Overall, it was observed that administration of vitamin C also inhibited inflammation and the infiltration of related inflammatory cells associated with BLM-induced pulmonary fibrosis. However, it was found to be more effectively inhibited in the group administered Aptamin C. Based on these findings, it can be concluded that Aptamin C effectively inhibits the progression of pulmonary fibrosis symptoms, associated inflammatory responses, and the fibrosis process in a BLM-induced pulmonary fibrosis animal model.

### 2.3. Aptamin C Increases the Expression of SVCT-1 in Cells and Lung Tissues

**Figure 4:**
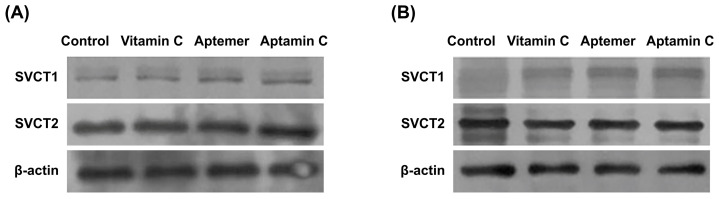
Increased SVCT-1 expression after Aptamin C treatment. HaCaT cells were treated with Aptamin C for 24 h. Aptamin C-treated HaCaT (**A**) and lung tissues (**B**) from mice were lysed and prepared for Western blot analysis. Proteins were separated on polyacrylamide-SDS gel and transferred to nitrocellulose membranes. Membranes were probed with anti-SVCT-1 and anti-SVCT-2. The same membranes were probed with β-actin antibody.

To examine the mechanism regarding the inhibition of symptoms and associated inflammatory responses of BLM-induced pulmonary fibrosis by Aptamin C, changes in the expression of Sodium-dependent Vitamin C Transporter-1/2 (SVCT-1/2), the specific channel proteins involved in the absorption of vitamin C into cells or tissues, were examined. Initially, the human keratinocyte cell line HaCaT was treated with vitamin C-specific aptamers, vitamin C, and Aptamin C, respectively. After 24 h, an increase in the expression of SVCT-1 was observed as a result of aptamer treatment. This increase was not observed in the group treated with vitamin C alone but was similarly observed in the group treated with Aptamin C (Figure 4A). Using lung tissue from a pulmonary fibrosis animal model induced by BLM, as used in the experiments described in Figure 3, changes in the expression of SVCT-1/2 were also examined. An increase in SVCT-1 expression was confirmed in lung tissues treated with aptamers and Aptamin C for 2 weeks after BLM treatment (Figure 4B). These findings imply that the aptamers used in Aptamin C, a complex with vitamin C, induce an increase in the expression of SVCT-1, a specific absorption channel for vitamin C, thereby enhancing the absorption rate of vitamin C.

## 3. Discussion

Pulmonary fibrosis is a disease in which lung tissue is damaged, becoming hardened and thickened, leading to serious respiratory function problems. The primary treatments for pulmonary fibrosis aim to reduce inflammatory responses and suppress immune reactions, using oral prednisone and injectable methylprednisolone [38]. However, long-term administration of steroid medications is known to cause side effects, including (1) weight gain and edema, (2) high blood pressure, (3) increased blood sugar, (4) osteoporosis, (5) weakened immune system, (6) skin issues, and (7) psychiatric side effects [39,40]. These side effects can severely impact patients’ quality of life, leading to increased treatment discontinuation rates, reduced adherence to therapy, and additional health complications. For example, weight gain and edema can restrict mobility, while osteoporosis increases fracture risk, particularly in older patients. Immune suppression increases susceptibility to infections, complicating pulmonary fibrosis management, and psychiatric effects can contribute to anxiety and depression, further diminishing the patient’s quality of life. In this respect, there is a need for therapies that manage the disease with fewer adverse impacts [40,41,42]. Recently, antifibrotic agents like pirfenidone and nintedanib have emerged, which offer symptom relief and slow disease progression, but their high cost remains a barrier. In this regard, this study aims to investigate the potential of Aptamin C as an adjunctive therapy for pulmonary fibrosis.

As shown in the results, Aptamin C increased the survival rate of the animal model of pulmonary fibrosis induced by BLM (Figure 1A), inhibited the infiltration of inflammatory cells into the lungs (Figure 2), and effectively suppressed the inflammatory and fibrotic responses in the lungs (Figure 3). Therefore, it is suggested that Aptamin C can be an effective adjunctive therapy for the onset and symptom relief of pulmonary fibrosis. Although the results were less significant compared to those observed in mice treated with Aptamin C, the fact that vitamin C also increased the survival rate of mice with induced pulmonary fibrosis indicates that the anti-inflammatory action based on the antioxidant efficacy of vitamin C played an important role in inhibiting the onset and progression of pulmonary fibrosis. This suggests that Aptamin C, which delays the oxidation of vitamin C using a vitamin C-specific aptamer, could be more effective against pulmonary fibrosis induced by BLM compared to vitamin C itself. In this study, it is confirmed that treatment of Aptamin C increases the expression of vitamin C transport more than vitamin C (Figure 4). In addition, previous research has shown that Aptamin C more effectively suppresses the pro-inflammatory cytokines IL-1α and IL-6 than vitamin C [24], demonstrating that it may play a role in managing inflammation associated with pulmonary fibrosis. Moreover, Aptamin C alleviates oxidative stress more than treatment with vitamin C alone [43]. This suggests that oxidative stress, which contributes to lung tissue damage and fibrosis progression, could be mitigated. Recent studies have shown that Aptamin C also enhances the efficacy of vitamin C in anti-inflammatory and anti-oxidative stress properties [24,32,43,44,45,46]. Therefore, we regard Aptamin C as a superior alternative to vitamin C to reduce fibrosis symptoms.

In the results related to the suppression of immune cell infiltration within the BALF as shown in Figure 2, we can see that a significant number of red blood cells exist in the alveolar fluid in the group that was administered BLM alone without vitamin C and Aptamin C (marked as CTL) and in the group that was administered aptamers only as a control for Aptamin C (marked as Aptamer). However, in the group that was administered both vitamin C and Aptamin C, red blood cells are almost invisible, and it can be confirmed that activated lymphocytes mainly exist when looking at the shape of the nucleus. This means that if vitamin C is not supplied for a considerable period, either in the form of vitamin C or Aptamin C, it leads to problems with the integrity of the vascular wall (especially the capillaries constituting the alveolar wall), resulting in vascular damage. Therefore, it implies that in patients with pulmonary fibrosis, if vitamin C is supplied either in the form of vitamin C or Aptamin C, it could maintain the function of the lungs through the structural maintenance of the vessels. This fact suggests that using vitamin C (especially in the form of Aptamin C, which enhances the efficacy of vitamin C) along with existing steroid formulations or antifibrotic agents could be expected to inhibit the occurrence and progression of inflammation mechanisms due to pulmonary fibrosis, as well as to maintain the structure of the lungs through the maintenance of vascular integrity.

The findings of this study collectively show the potential of Aptamin C as an effective adjunctive therapy for pulmonary fibrosis. However, as the evidence primarily relies on animal studies, it is crucial to conduct human clinical trials to validate these findings and collect data on the adjunctive efficacy of Aptamin C in pulmonary fibrosis. A careful review of Aptamin C’s clinical use from various perspectives is also essential to ensure its safety and effectiveness in practice.

Nevertheless, Aptamin C is anticipated to offer notable clinical benefits as an adjunctive therapy for pulmonary fibrosis. By potentially reducing the need for higher doses of steroids, it may minimize the associated side effects, thereby improving treatment tolerability and patient adherence. This study suggests that incorporating Aptamin C into treatment regimens for pulmonary fibrosis could not only slow disease progression but also improve overall patient outcomes by addressing both the underlying pathology and the adverse effects associated with current therapies. Not only with steroids, Aptamin C may also elicit a synergetic effect with existing antifibrotic medications. For instance, pirfenidone exerts its antifibrotic effects by modulating multiple pathways involved in fibrosis and inflammation, specifically by inhibiting pro-inflammatory cytokines such as IL-6 and TNF-α [47]. Previous docking analyses have shown that vitamin C can bind to the pirfenidone binding sites of IL-6 and TNF-α, suggesting potential inhibition of these cytokines [8]. Nintedanib works by inhibiting tyrosine kinases involved in fibrosis pathways, and Aptamin C may enhance its effects by addressing oxidative damage and inflammation, leading to a more comprehensive approach to managing fibrosis [48]. This combination could offer synergistic benefits not only in slowing disease progression but also in improving overall patient quality of life by enhanced management of inflammation and oxidative stress. However, limited data exist on direct interactions between Aptamin C and these antifibrotic drugs, and it is crucial to evaluate any potential pharmacokinetic or pharmacodynamic interactions. Increased antioxidant activity from Aptamin C could theoretically influence the metabolism of these drugs, necessitating further research to assess any potential risk.

In humans and primates, the L-gulono-γ-lactone oxidase (Gulo) gene is deficient, and endogenous vitamin C cannot be synthesized by the animals themselves [33,49]. However, other animals, especially mice commonly used in animal experiments, can synthesize large amounts of vitamin C within their bodies, which presents a challenge in accurately measuring the efficacy of externally administered vitamin C in animal studies. Therefore, the results of this study, which used Gulo KO mice (that like humans, lack the Gulo gene and cannot synthesize vitamin C endogenously) to create a pulmonary fibrosis model induced by BLM and then test the efficacy of Aptamin C, are very significant. In other words, the findings of our study demonstrate the effects of externally administered vitamin C and Aptamin C on the prevention and progression of pulmonary fibrosis in animals that have a very similar physiological environment to humans in terms of vitamin C biosynthesis.

Aptamers are single-stranded DNA or RNA oligonucleotides with high affinity and specificity for their target molecules [50]. Aptamers are known to effectively induce the activation of the immune system within the body, especially through the activation of the innate immune system via TLRs [51]. Therefore, while this study used aptamers to increase the stability of vitamin C, it was also important to verify any other molecular mechanisms involved. As shown in Figure 4, it was confirmed that treating with aptamers used for Aptamin C effectively increased the expression of Sodium-dependent Vitamin C Transporter 1/2 (SVCT-1/2), especially SVCT-1. Although SVCT-1/2 are structurally similar, they differ in expression locations and functional roles. SVCT-1 is primarily expressed in tissues such as the liver, kidneys, and intestines, responsible for the absorption and reabsorption processes of vitamin C in these tissues [10,52]. In contrast, SVCT-2 is expressed in tissues like the brain, eyes, heart, and lungs, with particularly important expression in the brain and retina, where maintaining high intracellular concentrations of vitamin C is necessary [53,54,55]. In lung tissue, the expression of SVCT-2 is primarily observed [52]. The lungs are one of the main antioxidant defense lines of the body, protecting against various toxins and pollutants from the external environment. The efficient intracellular transport of vitamin C via SVCT-2 strengthens the protective function against oxidative stress in lung cells and plays a crucial role in regulating inflammatory responses [33,56].

Therefore, the vitamin C-specific aptamer is thought to not only increase the stability and duration of vitamin C within the body, but also act on lung tissue to increase the expression of SVCT-1, thus enabling the absorption of additional amounts of vitamin C beyond what is absorbed through the already highly expressed SVCT-2. In addition to the action in lung tissue, a mechanism that activates STAT3 in NK cells has also been confirmed in studies targeting Gulo KO mice and human NK cells [37]. Therefore, the mechanisms of action in general body tissues and immune tissues may differ, and related further studies are currently underway.

Vitamin C is a substance produced during the metabolism of glucose in the liver and must be ingested through food or supplements [57,58]. The World Health Organization (WHO) recommends a daily intake of 90 mg of vitamin C for adult men and 75 mg for women [59]. However, pregnant or breastfeeding women, smokers, and people with various diseases may need more vitamin C [60,61]. Our research has shown that vitamin C has direct anticancer toxicity and effective antiviral efficacy, including against the influenza virus. It has also been reported to have anti-inflammatory efficacy against various inflammatory diseases, including hepatitis, IBD, myocarditis, degenerative inflammatory brain diseases, and skin inflammation diseases caused by UV irradiation [10,62,63,64]. Most importantly, it has been confirmed to have an excellent effect on activating immune functions, including NK cell action [10]. Although there is still controversy regarding the efficacy and action of vitamin C in the prevention and treatment of diseases, based on the positive aspects of vitamin C, Aptamin C is considered to be a more versatile substance in the prevention and treatment of diseases and activation of the immune system than vitamin C itself.

## 4. Materials and Methods

### 4.1. Cell Culture

Human keratinocyte cell line HaCaT was provided by Dr. N.E. Fusenig, DKFZ, Heidelberg, Germany. HaCaT was cultured in RPMI 1640 medium (WELGENE, Kyungsan, Republic of Korea) supplemented with 10% fetal bovine serum (Gibco, Grand Island, NY, USA), penicillin (100 U/mL; WELGENE), and streptomycin (100 µg/mL; WELGENE) at 37 °C in a humidified 5% CO_2_ incubator.

### 4.2. Aptamin C Preparation

A single-stranded DNA (ssDNA) aptamer that selectively binds to vitamin C was obtained from Nexmos Inc. The ssDNA aptamer library, containing random nucleotide sequences consisting of 30 nucleotides flanked by primer sites for amplification (5′-ATGCGGATCCCGCGC-(30 nucleotides)-GCGCAAGCTTCGCGC-3′), was screened using the SELEX (Systematic Evolution of Ligands by Exponential Enrichment) method with graphene oxide assistance (rGO-SELEX). After five rounds of enrichment, 119 sequences were obtained and grouped into 11 categories based on structural similarity. From these, a single representative sequence was selected and modified to a 20-mer as the vitamin C-specific aptamer. Aptamers were dissolved in PBS containing 1 mM of MgCl_2_ and heated for 30 min at 60 °C and then cooled. For in vitro experiments, L-ascorbic acid (Sigma-Aldrich, St. Louis, MO, USA) was added to the aptamer in a ratio of 1:100 (*w*/*w*) and the mixture was allowed to sit for 30 min at room temperature to enable the aptamers to fold into their tertiary structures. For the animal experiments, L-ascorbic acid was added to the aptamer in a ratio of 1:50 (*w*/*w*). Aptamin C was stored at 4 °C.

### 4.3. Mouse and BLM-Induced Fibrosis Animal Model

Gulo (−/−) knockout (KO) mice (Strain: C57BL/6) were housed under specific pathogen-free conditions at the animal facility of the Seoul National University College of Medicine. The KO mice were maintained with the supplementation of vitamin C (3.3 g/L) in their drinking water to prevent death caused by vitamin C deficiency. Before the generation of pulmonary fibrosis in KO mice, vitamin C supplementation was discontinued for 3 weeks, and then the mice (male, 11 weeks old) body weights were measured. The mice, whose average weight was 31.7 g, were randomly divided into five groups, and four groups received an intratracheal injection of bleomycin (1 mg/kg/30 µL; Sigma-Aldrich) dissolved in phosphate-buffered saline (PBS). Negative control group mice were intratracheally injected with an equal volume of PBS. Six hours after bleomycin injection, the mice were randomly divided into four groups (*n* = 5): (1) control, (2) vitamin C (100 mg/kg), (3) aptamer (2 mg/kg), and (4) Aptamin C (100 mg/kg vitamin C and 2 mg/kg aptamer), and each drug was intraperitoneally administered every day for 2 weeks. The weight of the mice was recorded three times per week for 2 weeks, and the mice were sacrificed on day 14. The tissue index was calculated as follows: tissue index = tissue weight (mg)/body weight (g). Animal experiments were reviewed and approved by the Institutional Animal Care and Use Committee of the Seoul National University College of Medicine animal facility (IACUC no. SNU-230601-2-3).

### 4.4. Survival Study

For the assessment of survival rates, wild-type mice were given 1.5 mg/kg bleomycin by intratracheal inoculation on day 1 and daily treated with vitamin C, aptamer, or Aptamin C (*n* = 8). The mortality of mice was monitored for 38 days.

### 4.5. Bronchoalveolar Lavage Fluid (BALF) Analysis

To obtain bronchoalveolar lavage fluid, the mice were euthanized and the trachea was cannulated with a 22-gauge intravenous catheter (BD Biosciences, Franklin Lakes, NJ, USA). The left lung was tied with a hemostat and the right lung was lavaged with 0.8 mL PBS by instillation and aspiration twice. The collected samples were centrifuged at 1400× *g* for 10 min at 4 °C and then the supernatants were removed. The pellet was resuspended in 400 µL PBS and 100 µL aliquots were spun onto a slide with a cytospin centrifuge (Thermo Fisher Scientific, Waltham MA, USA). The slides were stained with Wright–Giemsa (Sigma-Aldrich) to visualize immune cells.

### 4.6. Histological Analysis

The lung tissues were excised from mice and were fixed in 4% paraformaldehyde. Fixed tissues were placed in a tissue fixation cassette, and then dehydrated to enable complete penetration of the lung tissues by paraffin. The hydration was performed, as the concentration of alcohol increased, to replace the water in the tissue with alcohol. After dehydration, tissues were embedded in paraffin blocks. The block was sectioned into 4 µm thick slices. The slices were stained with hematoxylin and eosin (H&E; Sigma-Aldrich) or Masson’s trichrome staining (VitroVivo Biotech, Rockville, MD, USA) according to the manufacturer’s instructions. The stained tissue was visualized using a microscope (EVOS M5000, Invitrogen, Carlsbad, CA, USA). Microscopic lung fibrosis in H&E-stained lungs was scored using the Ashcroft scale 31. The severity of fibrosis in Masson’s trichrome-stained lungs was quantified by blue-stained area indicating collagen deposition using Celleste™ 5 software (Invitrogen).

### 4.7. Western Blot Analysis

HaCaT cells at a density of 1 × 10^6^ cells/mL cells in 6-well plates were cultured with vitamin C (0.33 mg/mL), aptamer (0.0033 mg/mL) and Aptamin C (a mixture of vitamin C (0.33 mg/mL) with aptamer (0.0033 g/mL) in a ratio of 1:100 (*w*/*w*)) for 24 h. HaCaT cells treated with Aptamin C and lung tissues from a pulmonary fibrosis mice model induced by BLM were lysed and proteins were extracted in lysis buffer containing 50 mM Tris-HCl (pH 7.4), 1% NP-40, 0.25% sodium deoxycholate, 150 mM NaCl, 1 mM EDTA, protease inhibitor, and phosphatase inhibitor cocktails (Sigma-Aldrich). Protein was mixed with 5x SDS sample buffer, and 20 μg of protein was separated onto 10% polyacrylamide-SDS gel. Separated proteins were transferred onto a nitrocellulose membrane and blocked with 5% skim milk solution in PBS containing 0.05% Tween 20 (PBS-T). The membrane was exposed to primary antibodies (goat anti-SVCT-1 and SVCT-2 antibody; 1:100; Santa Cruz Biotechnology, Dallas, TX, USA) at 4 °C overnight, followed by exposure to donkey-developed anti-Goat IgG biotinylated antibody (1:5000; Cell signaling) at room temperature for 1 h. The immunoreactive proteins were visualized with an enhanced chemiluminescence (Amersham) detection system (EZ-Western Lumi La; Dogen, Seoul, Republic of Korea).

### 4.8. Statistical Analysis

For all animal experiments, data are presented as the mean ± standard error of the mean (SEM). One-way analysis of variance (ANOVA) was performed when multiple groups were compared. A log-rank (Mantel–Cox) test was performed on the survival plots. Statistical analysis was performed using Prism 8.0 software (GraphPad Software, La Jolla, CA, USA).

## 5. Conclusions

This study investigates the potential of Aptamin C, a complex of vitamin C, and its specific aptamer in a mouse model of BLM-induced pulmonary fibrosis. Aptamin C demonstrated a superior effect in inhibiting the progression of BLM-induced pulmonary fibrosis and led to prolonged survival compared to vitamin C treatment alone. Mice supplemented with Aptamin C exhibited a significant reduction in inflammatory infiltration in BALF and markedly fewer fibrotic lesions in lung tissues than those treated with vitamin C. Additionally, Aptamin C treatment was observed to induce the expression of SVCT-1, a vitamin C transporter, in both human epithelial keratinocytes and mouse lung tissues. Collectively, these findings suggest that Aptamin C not only enhances the stability of vitamin C but also increases SVCT-1 expression, resulting in improved vitamin C absorption into cells and tissues. This dual effect contributes to the inhibition of symptom progression and associated inflammatory responses in pulmonary fibrosis.

## Data Availability

All data generated for this study are included in the manuscript.
